# 3-Acetyl-6-chloro-2-methyl-4-phenyl­quinolinium hydrogen sulfate

**DOI:** 10.1107/S1600536809048934

**Published:** 2009-11-21

**Authors:** Wan-Sin Loh, Hoong-Kun Fun, S. Sarveswari, V. Vijayakumar, B. Palakshi Reddy

**Affiliations:** a X-ray Crystallography Unit, School of Physics, Universiti Sains Malaysia, 11800 USM, Penang, Malaysia; b Organic Chemistry Division, School of Science, VIT University, Vellore-632 014, India

## Abstract

In the title salt, C_18_H_15_ClNO^+^·HSO_4_^−^, the quinolinium ring system is approximately planar, with a maximum deviation of 0.028 (2) Å, and forms a dihedral angle of 78.43 (4)° with the attached phenyl ring. A pair of inter­molecular O—H⋯O hydrogen bonds links two hydrogen sulfate anions into a dimer, generating a *R*_2_^2^(8) ring motif. Inter­molecular N—H⋯O hydrogen bonds and C—H⋯O contacts link the ions into a three-dimensional network. The structure is further stabilized by C—H⋯π inter­actions

## Related literature

For the background to and biological activities of quinolines, see: Morimoto *et al.* (1991 ▶); Michael (1997 ▶); Markees *et al.* (1970 ▶); Campbell *et al.* (1988 ▶); Maguire *et al.* (1994 ▶); Kalluraya & Sreenivasa (1998 ▶); Roma *et al.* (2000 ▶); Chen *et al.* (2001 ▶). For related structure: see: Fun *et al.* (2009 ▶). For hydrogen bond motifs, see: Bernstein *et al.* (1995 ▶). For the stability of the temperature controller used for the data collection, see: Cosier & Glazer (1986 ▶).
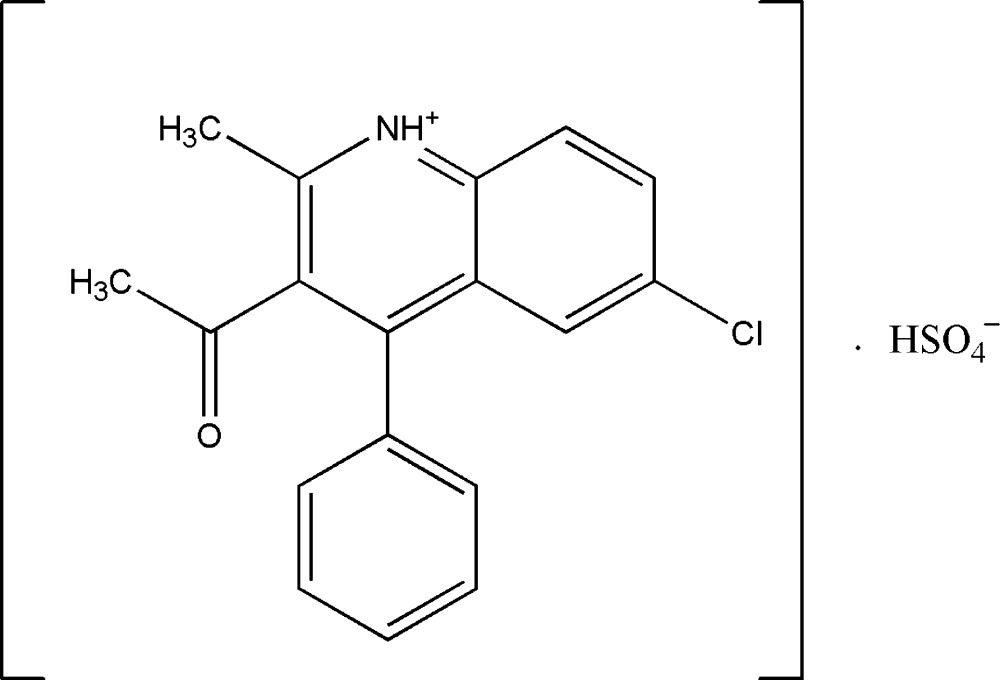


## Experimental

### 

#### Crystal data

C_18_H_15_ClNO^+^·HSO_4_^−^
*M* *_r_* = 393.83
Triclinic, 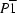

*a* = 7.3912 (1) Å
*b* = 8.8547 (1) Å
*c* = 13.3413 (2) Å
α = 92.485 (1)°
β = 91.889 (1)°
γ = 99.539 (1)°
*V* = 859.55 (2) Å^3^
*Z* = 2
Mo *K*α radiation
μ = 0.37 mm^−1^
*T* = 100 K
0.28 × 0.18 × 0.11 mm

#### Data collection

Bruker SMART APEXII CCD area-detector diffractometer
Absorption correction: multi-scan (**SADABS**; Bruker, 2005 ▶) *T*_min_ = 0.902, *T*_max_ = 0.960
20789 measured reflections
5036 independent reflections
4099 reflections with *I* > 2σ(*I*)
*R*_int_ = 0.036

#### Refinement

*R*[*F* ^2^ > 2σ(*F* ^2^)] = 0.044
*wR*(*F* ^2^) = 0.100
*S* = 1.05
5036 reflections
299 parameters
All H-atom parameters refined
Δρ_max_ = 0.49 e Å^−3^
Δρ_min_ = −0.46 e Å^−3^

### 

Data collection: *APEX2* (Bruker, 2005 ▶); cell refinement: *SAINT* (Bruker, 2005 ▶); data reduction: *SAINT*; program(s) used to solve structure: *SHELXTL* (Sheldrick, 2008 ▶); program(s) used to refine structure: *SHELXTL*; molecular graphics: *SHELXTL*; software used to prepare material for publication: *SHELXTL* and *PLATON* (Spek, 2009 ▶).

## Supplementary Material

Crystal structure: contains datablocks global, I. DOI: 10.1107/S1600536809048934/tk2576sup1.cif

Structure factors: contains datablocks I. DOI: 10.1107/S1600536809048934/tk2576Isup2.hkl

Additional supplementary materials:  crystallographic information; 3D view; checkCIF report

## Figures and Tables

**Table 1:** Hydrogen-bond geometry (Å, °)

*D*—H⋯*A*	*D*—H	H⋯*A*	*D*⋯*A*	*D*—H⋯*A*
N1—H1*N*1⋯O5^i^	0.88 (2)	1.86 (2)	2.7200 (19)	168 (2)
O2—H1*O*2⋯O3^ii^	0.77 (4)	1.84 (4)	2.6027 (19)	180 (5)
C5—H5*A*⋯O3^iii^	0.96 (2)	2.58 (2)	3.304 (2)	132.5 (17)
C15—H15*A*⋯O4	0.93 (2)	2.55 (2)	3.381 (2)	148.0 (15)
C16—H16*C*⋯O4^iv^	0.95 (3)	2.55 (3)	3.332 (2)	139 (2)
C12—H12*A*⋯*Cg*1^v^	0.95 (2)	2.74 (2)	3.5884 (18)	149.1 (14)

Symmetry codes: (i) 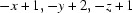; (ii) 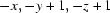; (iii) 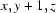; (iv) 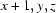; (v) 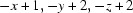. *Cg*1 is the centroid of the C1–C6 ring.

**Fig. 1.**
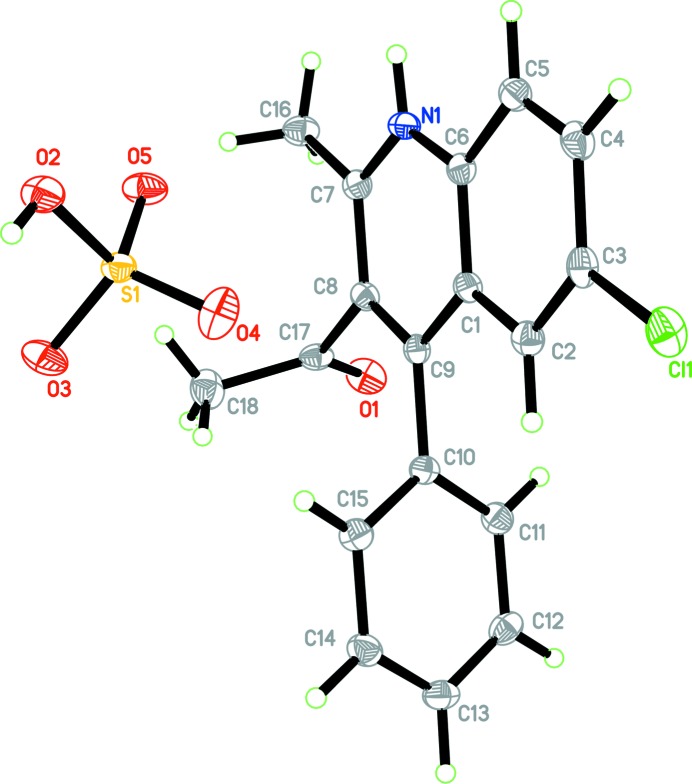
The molecular structure of (I), showing 30% probability displacement ellipsoids and the atom-numbering scheme.

**Fig. 2.**
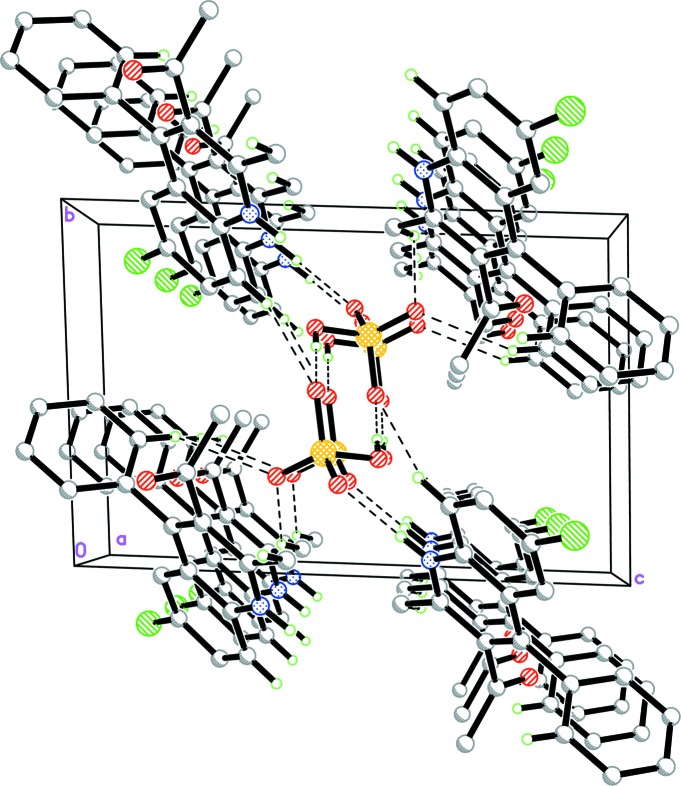
The crystal packing of (I), viewed along the a axis, showing the 3-D network. H atoms not involved in the intermolecular interactions (dashed lines) have been omitted for clarity.

## supplementary crystallographic information

### Comment

Quinolines and their derivatives are very important compounds because of their wide occurrence in natural products (Morimoto *et al.*, 1991; Michael, 1997) and biologically active compounds (Markees *et al.*, 1970; Campbell *et al.*, 1988). A large variety of quinolines have interesting physiological activities and have found attractive applications as pharmaceuticals, agrochemicals and as synthetic building blocks (Maguire *et al.*, 1994; Kalluraya & Sreenivasa, 1998; Roma *et al.*, 2000; Chen *et al.*, 2001). The 3-acetyl-6-chloro-2-methyl-4-phenylquinoline has been synthesized using the method available in the literature (Fun *et al.*, 2009), and then converted into the title salt, (I).

The asymmetric unit of (I), Fig. 1, contains a 3-acetyl-6-chloro-2-methyl-4-phenylquinolinium cation and a hydrogen sulfate anion. One proton is transferred from the hydroxyl group of hydrogen sulfate to the atom N1 of 3-acetyl-6-chloro-2-methyl-4-phenylquinoline during crystallisation resulting in the formation of salt, (I). The quinolinium ring system (C1–C9/N1) is approximately planar with a maximum deviation of 0.028 (2) Å at atom C7. This mean plane of the quinolinium ring forms a dihedral angle of 78.43 (4)° with the phenyl ring (C10–C15). Bond lengths and angles are comparable to a closely related structure (Fun *et al.*, 2009).

In the crystal packing (Fig. 2), a pair of O2—H1O2···O3 hydrogen bonds link two hydrogen sulfate anions into dimers, generating *R*_2_^2^(8) ring motifs stacked along *a* axis (Bernstein *et al.*, 1995). A N1—H1N1—O5 hydrogen bond links the dimer with the quinolinium ring system. The ions are linked into a 3-D network by C5—H5A···O3, C15—H15A···O4 and C16—H16C···O4 contacts. The structure is further stabilized by C—H···π interactions (Table 1), involving C1–C6 (centroid *Cg*1) ring system.

### Experimental

To a solution of 3-acetyl-6-chloro-2-methyl-4-phenylquinoline (10 ml, 1 *M*) in ethanol, copper sulfate solution (1 ml, 1 *M*) and concentrated H_2_SO_4_ (1 ml) was added and then refluxed for about 10 min. The contents were filtered and kept for 92 h for crystallization.

### Refinement

All hydrogen atoms were located from the difference Fourier map and were refined freely (range of C–H = 0.91 (3) - 0.96 (3) Å and see Table 1).

### Crystal data

**Table:**

C_18_H_15_ClNO^+^·HSO_4_^−^	*Z* = 2
*M**_r_* = 393.83	*F*(000) = 408
Triclinic, *P*1	*D*_x_ = 1.522 Mg m^−^^3^
Hall symbol: -P 1	Mo *K*α radiation, λ = 0.71073 Å
*a* = 7.3912 (1) Å	Cell parameters from 6943 reflections
*b* = 8.8547 (1) Å	θ = 2.3–30.0°
*c* = 13.3413 (2) Å	µ = 0.37 mm^−^^1^
α = 92.485 (1)°	*T* = 100 K
β = 91.889 (1)°	Block, colourless
γ = 99.539 (1)°	0.28 × 0.18 × 0.11 mm
*V* = 859.55 (2) Å^3^	

### Data collection

**Table:**

Bruker SMART APEXII CCD area-detector diffractometer	5036 independent reflections
Radiation source: fine-focus sealed tube	4099 reflections with *I* > 2σ(*I*)
graphite	*R*_int_ = 0.036
φ and ω scans	θ_max_ = 30.1°, θ_min_ = 2.3°
Absorption correction: multi-scan (*SADABS*; Bruker, 2005)	*h* = −10→10
*T*_min_ = 0.902, *T*_max_ = 0.960	*k* = −12→12
20789 measured reflections	*l* = −17→18

### Refinement

**Table:**

Refinement on *F*^2^	Primary atom site location: structure-invariant direct methods
Least-squares matrix: full	Secondary atom site location: difference Fourier map
*R*[*F*^2^ > 2σ(*F*^2^)] = 0.044	Hydrogen site location: inferred from neighbouring sites
*wR*(*F*^2^) = 0.100	All H-atom parameters refined
*S* = 1.05	*w* = 1/[σ^2^(*F*_o_^2^) + (0.0423*P*)^2^ + 0.5203*P*] where *P* = (*F*_o_^2^ + 2*F*_c_^2^)/3
5036 reflections	(Δ/σ)_max_ < 0.001
299 parameters	Δρ_max_ = 0.49 e Å^−^^3^
0 restraints	Δρ_min_ = −0.46 e Å^−^^3^

### Special details

**Table:**

Experimental. The crystal was placed in the cold stream of an Oxford Cyrosystems Cobra open-flow nitrogen cryostat (Cosier & Glazer, 1986) operating at 100.0 (1) K.
Geometry. All e.s.d.'s (except the e.s.d. in the dihedral angle between two l.s. planes) are estimated using the full covariance matrix. The cell e.s.d.'s are taken into account individually in the estimation of e.s.d.'s in distances, angles and torsion angles; correlations between e.s.d.'s in cell parameters are only used when they are defined by crystal symmetry. An approximate (isotropic) treatment of cell e.s.d.'s is used for estimating e.s.d.'s involving l.s. planes.
Refinement. Refinement of *F*^2^ against ALL reflections. The weighted *R*-factor *wR* and goodness of fit *S* are based on *F*^2^, conventional *R*-factors *R* are based on *F*, with *F* set to zero for negative *F*^2^. The threshold expression of *F*^2^ > σ(*F*^2^) is used only for calculating *R*-factors(gt) *etc*. and is not relevant to the choice of reflections for refinement. *R*-factors based on *F*^2^ are statistically about twice as large as those based on *F*, and *R*- factors based on ALL data will be even larger.

### Fractional atomic coordinates and isotropic or equivalent isotropic displacement parameters (Å^2^)

**Table:**

	*x*	*y*	*z*	*U*_iso_*/*U*_eq_	
Cl1	−0.07965 (6)	1.15710 (5)	0.87223 (3)	0.02195 (11)	
O1	0.84858 (17)	0.74796 (14)	0.81500 (10)	0.0227 (3)	
N1	0.56245 (19)	1.06536 (16)	0.64462 (11)	0.0144 (3)	
C1	0.3576 (2)	0.98834 (18)	0.77580 (12)	0.0130 (3)	
C2	0.2022 (2)	1.01001 (19)	0.83015 (13)	0.0153 (3)	
C3	0.1135 (2)	1.12951 (19)	0.80771 (13)	0.0161 (3)	
C4	0.1734 (2)	1.23192 (19)	0.73288 (13)	0.0174 (3)	
C5	0.3223 (2)	1.21203 (19)	0.67852 (13)	0.0163 (3)	
C6	0.4145 (2)	1.08892 (18)	0.69952 (12)	0.0138 (3)	
C7	0.6577 (2)	0.95245 (18)	0.65899 (12)	0.0144 (3)	
C8	0.6082 (2)	0.85283 (17)	0.73720 (12)	0.0135 (3)	
C9	0.4607 (2)	0.86836 (17)	0.79479 (12)	0.0128 (3)	
C10	0.4060 (2)	0.76276 (17)	0.87655 (12)	0.0131 (3)	
C11	0.5030 (2)	0.78222 (19)	0.96875 (13)	0.0165 (3)	
C12	0.4476 (2)	0.6859 (2)	1.04564 (13)	0.0178 (3)	
C13	0.2988 (2)	0.56871 (19)	1.02989 (14)	0.0184 (3)	
C14	0.2033 (2)	0.54837 (19)	0.93779 (14)	0.0185 (3)	
C15	0.2549 (2)	0.64550 (19)	0.86090 (13)	0.0163 (3)	
C16	0.8098 (2)	0.9359 (2)	0.59146 (14)	0.0191 (3)	
C17	0.7189 (2)	0.72549 (19)	0.75589 (13)	0.0157 (3)	
C18	0.6537 (3)	0.5756 (2)	0.69949 (16)	0.0243 (4)	
S1	0.21895 (5)	0.66550 (5)	0.53846 (3)	0.01523 (10)	
O3	0.22537 (17)	0.50022 (14)	0.54897 (10)	0.0226 (3)	
O2	0.09255 (19)	0.67613 (15)	0.44494 (10)	0.0218 (3)	
O4	0.14599 (19)	0.73228 (17)	0.62452 (10)	0.0284 (3)	
O5	0.39820 (17)	0.74590 (14)	0.51153 (10)	0.0217 (3)	
H2A	0.158 (3)	0.941 (2)	0.8811 (15)	0.014 (5)*	
H4A	0.108 (3)	1.313 (2)	0.7209 (16)	0.024 (5)*	
H5A	0.363 (3)	1.281 (2)	0.6270 (16)	0.023 (5)*	
H11A	0.606 (3)	0.861 (2)	0.9797 (16)	0.023 (5)*	
H12A	0.513 (3)	0.700 (2)	1.1086 (16)	0.019 (5)*	
H13A	0.258 (3)	0.501 (2)	1.0818 (17)	0.026 (6)*	
H14A	0.100 (3)	0.468 (2)	0.9267 (16)	0.025 (5)*	
H15A	0.189 (3)	0.634 (2)	0.7994 (15)	0.016 (5)*	
H18A	0.736 (3)	0.507 (3)	0.7149 (18)	0.038 (7)*	
H18B	0.536 (3)	0.538 (3)	0.7207 (18)	0.032 (6)*	
H18C	0.641 (3)	0.587 (3)	0.631 (2)	0.039 (7)*	
H16A	0.772 (4)	0.847 (3)	0.548 (2)	0.057 (8)*	
H16B	0.838 (4)	1.017 (3)	0.552 (2)	0.042 (7)*	
H16C	0.921 (4)	0.922 (3)	0.6250 (19)	0.039 (7)*	
H1N1	0.592 (3)	1.124 (3)	0.5945 (18)	0.032 (6)*	
H1O2	−0.001 (5)	0.624 (4)	0.447 (2)	0.070 (11)*	

### Atomic displacement parameters (Å^2^)

**Table:**

	*U*^11^	*U*^22^	*U*^33^	*U*^12^	*U*^13^	*U*^23^
Cl1	0.01539 (19)	0.0206 (2)	0.0306 (2)	0.00572 (15)	0.00463 (16)	−0.00319 (17)
O1	0.0157 (6)	0.0233 (6)	0.0296 (7)	0.0043 (5)	−0.0031 (5)	0.0052 (5)
N1	0.0154 (6)	0.0136 (6)	0.0140 (7)	0.0013 (5)	0.0007 (5)	0.0032 (5)
C1	0.0121 (7)	0.0123 (7)	0.0143 (7)	0.0013 (5)	−0.0003 (6)	−0.0004 (6)
C2	0.0130 (7)	0.0154 (7)	0.0168 (8)	0.0004 (6)	0.0000 (6)	−0.0004 (6)
C3	0.0126 (7)	0.0169 (7)	0.0186 (8)	0.0031 (6)	−0.0001 (6)	−0.0042 (6)
C4	0.0193 (8)	0.0147 (7)	0.0188 (8)	0.0057 (6)	−0.0043 (6)	−0.0009 (6)
C5	0.0185 (8)	0.0147 (7)	0.0155 (8)	0.0027 (6)	−0.0027 (6)	0.0003 (6)
C6	0.0140 (7)	0.0133 (7)	0.0136 (8)	0.0011 (6)	−0.0002 (6)	−0.0004 (6)
C7	0.0129 (7)	0.0148 (7)	0.0147 (8)	0.0004 (6)	−0.0006 (6)	0.0001 (6)
C8	0.0130 (7)	0.0124 (7)	0.0149 (8)	0.0015 (6)	−0.0005 (6)	0.0007 (6)
C9	0.0123 (7)	0.0123 (7)	0.0129 (7)	−0.0002 (6)	−0.0015 (6)	−0.0011 (6)
C10	0.0124 (7)	0.0128 (7)	0.0151 (8)	0.0042 (6)	0.0036 (6)	0.0017 (6)
C11	0.0152 (8)	0.0163 (7)	0.0175 (8)	0.0017 (6)	−0.0002 (6)	0.0006 (6)
C12	0.0189 (8)	0.0219 (8)	0.0142 (8)	0.0079 (6)	0.0006 (6)	0.0023 (6)
C13	0.0211 (8)	0.0168 (8)	0.0198 (9)	0.0078 (6)	0.0080 (7)	0.0056 (6)
C14	0.0171 (8)	0.0157 (8)	0.0221 (9)	0.0006 (6)	0.0055 (7)	0.0013 (6)
C15	0.0144 (7)	0.0180 (8)	0.0163 (8)	0.0017 (6)	0.0002 (6)	0.0014 (6)
C16	0.0170 (8)	0.0205 (8)	0.0202 (9)	0.0028 (7)	0.0050 (7)	0.0049 (7)
C17	0.0141 (7)	0.0175 (8)	0.0168 (8)	0.0043 (6)	0.0046 (6)	0.0052 (6)
C18	0.0292 (10)	0.0202 (9)	0.0253 (10)	0.0104 (8)	−0.0030 (8)	−0.0014 (7)
S1	0.01399 (19)	0.01650 (19)	0.0145 (2)	0.00016 (14)	−0.00041 (14)	0.00325 (14)
O3	0.0195 (6)	0.0172 (6)	0.0317 (7)	0.0035 (5)	−0.0023 (5)	0.0090 (5)
O2	0.0197 (6)	0.0224 (6)	0.0219 (7)	−0.0004 (5)	−0.0081 (5)	0.0066 (5)
O4	0.0267 (7)	0.0362 (8)	0.0224 (7)	0.0081 (6)	0.0001 (6)	−0.0061 (6)
O5	0.0152 (6)	0.0243 (6)	0.0239 (7)	−0.0029 (5)	−0.0023 (5)	0.0094 (5)

### Geometric parameters (Å, °)

**Table:**

Cl1—C3	1.7373 (17)	C11—C12	1.391 (2)
O1—C17	1.206 (2)	C11—H11A	0.95 (2)
N1—C7	1.332 (2)	C12—C13	1.385 (3)
N1—C6	1.375 (2)	C12—H12A	0.95 (2)
N1—H1N1	0.88 (2)	C13—C14	1.386 (3)
C1—C6	1.410 (2)	C13—H13A	0.96 (2)
C1—C2	1.414 (2)	C14—C15	1.390 (2)
C1—C9	1.433 (2)	C14—H14A	0.95 (2)
C2—C3	1.372 (2)	C15—H15A	0.93 (2)
C2—H2A	0.961 (19)	C16—H16A	0.96 (3)
C3—C4	1.410 (2)	C16—H16B	0.91 (3)
C4—C5	1.369 (2)	C16—H16C	0.95 (3)
C4—H4A	0.95 (2)	C17—C18	1.496 (3)
C5—C6	1.411 (2)	C18—H18A	0.95 (3)
C5—H5A	0.96 (2)	C18—H18B	0.94 (3)
C7—C8	1.414 (2)	C18—H18C	0.92 (3)
C7—C16	1.486 (2)	S1—O4	1.4306 (14)
C8—C9	1.376 (2)	S1—O5	1.4602 (13)
C8—C17	1.523 (2)	S1—O3	1.4846 (12)
C9—C10	1.490 (2)	S1—O2	1.5495 (13)
C10—C11	1.393 (2)	O2—H1O2	0.77 (3)
C10—C15	1.396 (2)		
			
C7—N1—C6	124.00 (14)	C10—C11—H11A	120.5 (13)
C7—N1—H1N1	117.5 (16)	C13—C12—C11	120.13 (16)
C6—N1—H1N1	118.4 (16)	C13—C12—H12A	120.1 (12)
C6—C1—C2	118.86 (14)	C11—C12—H12A	119.7 (13)
C6—C1—C9	118.26 (14)	C12—C13—C14	120.02 (16)
C2—C1—C9	122.87 (15)	C12—C13—H13A	121.6 (13)
C3—C2—C1	118.80 (15)	C14—C13—H13A	118.4 (14)
C3—C2—H2A	120.3 (12)	C13—C14—C15	120.51 (16)
C1—C2—H2A	120.9 (12)	C13—C14—H14A	120.4 (13)
C2—C3—C4	122.16 (16)	C15—C14—H14A	119.1 (13)
C2—C3—Cl1	119.71 (13)	C14—C15—C10	119.42 (16)
C4—C3—Cl1	118.13 (13)	C14—C15—H15A	121.0 (12)
C5—C4—C3	120.02 (15)	C10—C15—H15A	119.6 (12)
C5—C4—H4A	121.3 (13)	C7—C16—H16A	108.2 (18)
C3—C4—H4A	118.7 (13)	C7—C16—H16B	113.0 (17)
C4—C5—C6	118.85 (15)	H16A—C16—H16B	107 (2)
C4—C5—H5A	120.4 (13)	C7—C16—H16C	114.5 (15)
C6—C5—H5A	120.8 (13)	H16A—C16—H16C	107 (2)
N1—C6—C1	118.95 (14)	H16B—C16—H16C	107 (2)
N1—C6—C5	119.76 (14)	O1—C17—C18	123.85 (16)
C1—C6—C5	121.29 (15)	O1—C17—C8	119.92 (15)
N1—C7—C8	118.46 (15)	C18—C17—C8	116.21 (15)
N1—C7—C16	118.53 (15)	C17—C18—H18A	108.6 (15)
C8—C7—C16	123.01 (14)	C17—C18—H18B	107.4 (15)
C9—C8—C7	120.87 (14)	H18A—C18—H18B	111 (2)
C9—C8—C17	120.18 (14)	C17—C18—H18C	112.1 (15)
C7—C8—C17	118.94 (14)	H18A—C18—H18C	112 (2)
C8—C9—C1	119.40 (14)	H18B—C18—H18C	106 (2)
C8—C9—C10	121.29 (14)	O4—S1—O5	114.16 (8)
C1—C9—C10	119.31 (14)	O4—S1—O3	112.00 (8)
C11—C10—C15	120.07 (15)	O5—S1—O3	110.14 (8)
C11—C10—C9	120.25 (14)	O4—S1—O2	109.30 (8)
C15—C10—C9	119.68 (14)	O5—S1—O2	104.05 (8)
C12—C11—C10	119.83 (16)	O3—S1—O2	106.62 (7)
C12—C11—H11A	119.6 (13)	S1—O2—H1O2	112 (2)
			
C6—C1—C2—C3	−0.9 (2)	C7—C8—C9—C10	179.22 (15)
C9—C1—C2—C3	179.05 (15)	C17—C8—C9—C10	0.3 (2)
C1—C2—C3—C4	−0.5 (3)	C6—C1—C9—C8	−1.4 (2)
C1—C2—C3—Cl1	179.14 (12)	C2—C1—C9—C8	178.66 (15)
C2—C3—C4—C5	1.3 (3)	C6—C1—C9—C10	178.62 (14)
Cl1—C3—C4—C5	−178.37 (13)	C2—C1—C9—C10	−1.3 (2)
C3—C4—C5—C6	−0.6 (2)	C8—C9—C10—C11	78.3 (2)
C7—N1—C6—C1	−0.2 (2)	C1—C9—C10—C11	−101.73 (18)
C7—N1—C6—C5	−179.97 (15)	C8—C9—C10—C15	−102.73 (19)
C2—C1—C6—N1	−178.14 (14)	C1—C9—C10—C15	77.3 (2)
C9—C1—C6—N1	1.9 (2)	C15—C10—C11—C12	−0.9 (2)
C2—C1—C6—C5	1.6 (2)	C9—C10—C11—C12	178.09 (15)
C9—C1—C6—C5	−178.35 (15)	C10—C11—C12—C13	1.5 (3)
C4—C5—C6—N1	178.89 (15)	C11—C12—C13—C14	−0.8 (3)
C4—C5—C6—C1	−0.8 (2)	C12—C13—C14—C15	−0.4 (3)
C6—N1—C7—C8	−1.9 (2)	C13—C14—C15—C10	1.0 (3)
C6—N1—C7—C16	177.43 (15)	C11—C10—C15—C14	−0.3 (2)
N1—C7—C8—C9	2.4 (2)	C9—C10—C15—C14	−179.33 (15)
C16—C7—C8—C9	−176.91 (16)	C9—C8—C17—O1	−89.5 (2)
N1—C7—C8—C17	−178.63 (14)	C7—C8—C17—O1	91.6 (2)
C16—C7—C8—C17	2.0 (2)	C9—C8—C17—C18	89.0 (2)
C7—C8—C9—C1	−0.8 (2)	C7—C8—C17—C18	−89.93 (19)
C17—C8—C9—C1	−179.69 (14)		

### Hydrogen-bond geometry (Å, °)

**Table:**

*D*—H···*A*	*D*—H	H···*A*	*D*···*A*	*D*—H···*A*
N1—H1N1···O5^i^	0.88 (2)	1.86 (2)	2.7200 (19)	168 (2)
O2—H1O2···O3^ii^	0.77 (4)	1.84 (4)	2.6027 (19)	180 (5)
C5—H5A···O3^iii^	0.96 (2)	2.58 (2)	3.304 (2)	132.5 (17)
C15—H15A···O4	0.93 (2)	2.55 (2)	3.381 (2)	148.0 (15)
C16—H16C···O4^iv^	0.95 (3)	2.55 (3)	3.332 (2)	139 (2)
C12—H12A···Cg1^v^	0.95 (2)	2.74 (2)	3.5884 (18)	149.1 (14)

Symmetry codes: (i) −*x*+1, −*y*+2, −*z*+1; (ii) −*x*, −*y*+1, −*z*+1; (iii) *x*, *y*+1, *z*; (iv) *x*+1, *y*, *z*; (v) −*x*+1, −*y*+2, −*z*+2.

## References

[bb1] Bernstein, J., Davis, R. E., Shimoni, L. & Chang, N.-L. (1995). *Angew. Chem. Int. Ed. Engl.* **34**, 1555–1573.

[bb2] Bruker (2005). *APEX2*, *SAINT* and *SADABS*. Bruker AXS Inc., Madison, Wisconsin, USA.

[bb3] Campbell, S. F., Hardstone, J. D. & Palmer, M. J. (1988). *J. Med. Chem.* **31**, 1031–1035. 10.1021/jm00400a025 2896245

[bb4] Chen, Y.-L., Fang, K.-C., Sheu, J.-Y., Hsu, S.-L. & Tzeng, C.-C. (2001). *J. Med. Chem.* **44**, 2374–2377. 10.1021/jm0100335 11428933

[bb5] Cosier, J. & Glazer, A. M. (1986). *J. Appl. Cryst.* **19**, 105–107.

[bb6] Fun, H.-K., Loh, W.-S., Sarveswari, S., Vijayakumar, V. & Reddy, B. P. (2009). *Acta Cryst.* E**65**, o2688–o2689. 10.1107/S1600536809040306 PMC2971397 21578294

[bb7] Kalluraya, B. & Sreenivasa, S. (1998). *Il Farmaco*, **53**, 399–404. 10.1016/s0014-827x(98)00037-8 9764472

[bb8] Maguire, M. P., Sheets, K. R., McVety, K., Spada, A. P. & Zilberstein, A. (1994). *J. Med. Chem.* **37**, 2129–2137. 10.1021/jm00040a003 8035419

[bb9] Markees, D. G., Dewey, V. C. & Kidder, G. W. (1970). *J. Med. Chem.* **13**, 324–326. 10.1021/jm00296a048 5418519

[bb10] Michael, J. P. (1997). *Nat. Prod. Rep.* **14**, 605–608.

[bb11] Morimoto, Y., Matsuda, F. & Shirahama, H. (1991). *Synlett*, **3**, 202–203.

[bb12] Roma, G., Braccio, M. D., Grossi, G., Mattioli, F. & Ghia, M. (2000). *Eur. J. Med. Chem.* **35**, 1021–1026. 10.1016/s0223-5234(00)01175-2 11137230

[bb13] Sheldrick, G. M. (2008). *Acta Cryst.* A**64**, 112–122. 10.1107/S0108767307043930 18156677

[bb14] Spek, A. L. (2009). *Acta Cryst.* D**65**, 148–155. 10.1107/S090744490804362X PMC2631630 19171970

